# Towards molecular spintronics

**DOI:** 10.3762/bjnano.8.245

**Published:** 2017-11-21

**Authors:** Georgeta Salvan, Dietrich R T Zahn

**Affiliations:** 1 Physics Department, Semiconductor Physics, Technische Universität Chemnitz, Reichenhainer Straße 70, 09126 Chemnitz, Germany

**Keywords:** density functional theory, electrical and spin transport, Green’s function method, interfaces, magnetic molecules, (magneto-)optical spectroscopy, molecular spintronics, photoelectron spectroscopy, surface science, thin films

The discovery of tunneling and giant magnetoresistance in inorganic spin valves has led to a revolution in the field of magnetic memory and the significant increase in the storage capacity of modern hard drives. Simultaneously, given their inexpensive production, flexibility and diverse applications, molecular-based organic materials have become extremely important in electronic devices and circuitry. A combination of spintronics and organic electronics is expected to lead to a new generation of spin-based devices. These devices are expected to bring a wide range of exciting, new fields of application and products for organic/molecular spintronics.

The work of the Research Unit “Towards Molecular Spintronics” funded by the German Science Foundation (Deutsche Forschungsgemeinschaft, DFG) (DFG FOR 1154) was focused on the ultimate down-scaled functional unit which integrates the spintronic functionality into one single molecule. In our ambitious approach towards molecular spintronics we combined two interdisciplinary research fields based in otherwise disjunctive research communities: organic electronics and molecular magnetism. This required close collaboration of experts in physics (including theoretical physics), chemistry, materials science, and electrical engineering. The partners involved in this consortium are located at four Saxonian universities (Technische Universität Chemnitz, Technische Universität Dresden, Universität Leipzig, and Technische Universität Bergakademie Freiberg) as well as the Leibniz-Institut für Festkörper- und Werkstoff-Forschung (IFW) in Dresden, Germany.

The activities in this research unit were tailored to systematically address the whole research and development chain from molecule synthesis and molecular film deposition via fundamental characterization and theoretical understanding to device demonstration and integration.

**Tailoring and fundamental characterization of magnetic molecules:** Magnetic molecules for implementation into devices were synthesized and fundamentally investigated by theoretical density functional methods and experimental scanning probe techniques as well as optical and magnetic measurements on bulk materials. Already, at this stage, basic compatibility aspects concerning device processing had to be taken into account.

**Fabrication, characterization, and optimization of molecular thin films and interfaces:** Various deposition techniques to create suitable molecular films were tested for a variety of molecules. The structure, morphology, and molecular orientation of the layers were fundamentally characterized and optimized, taking into account that for device integration, the molecular layers need to obey certain boundary conditions, such as long-term stability, process compatibility, and the ability to integrate with electrode materials. Progress in this respect was only made possible by a continuous feedback from basic characterization and technology projects that allowed targeted and efficient synthesis of appropriate molecules and molecular films.

**Device demonstration and on-chip integration:** Rolled-up nanotechnology was used to create vertically stacked electronic devices with an organic tunnel transport layer for sensing applications. Horizontally stacked two-terminal and four-terminal devices photo sensors and magnetic field sensors were developed for large-scale integration purposes.

This complex research and development chain required various synthesis methods and theoretical approaches for prediction of molecular and devices properties. This included high-end and complementary characterization methods (ranging from static and dynamic magnetic characterization to local probe methods), as well as (magneto-)optical and electron spectroscopy, electrical measurements, new technological concepts, and sophisticated processing facilities. Such a complex chain can only become and remain successfully operational thanks to the enthusiastic cooperation between the groups, and in particular, between the young researchers. We would therefore like to express our deepest gratitude to all principal investigators, young researchers, associated researchers, and the coordinator (Jane Eisentraut) of the Research Unit for the great working atmosphere throughout the past six and a half years.

In this Thematic Series we summarize selected examples of the collaborative work in which at least two groups were involved. The range of considered molecules spans from heterotrinuclear bis(oxamato)-type and bis(oxamidato)-type complexes [1–3], to exchange-coupled dinickel complexes [4], metallo-phthalocyanines [5–7], metallo-porphyrins [8–9] and charge-transfer complexes [10–11], to metal-free molecules like pentacene-derivatives [12], fullerenes [13], trimesic acid [14], or organic ferromagnets [15]. Besides the internal cooperation, the Research Unit greatly profited from excellent talks and thorough discussions with external guests joining our scientific workshops and we are happy to host six articles from our invited guests with topics beyond the molecular systems investigated in our Research Unit, for example: theoretical predictions on metal/C_60_ interfaces [16], magneto-resistive donor/acceptor transistors [17], spin-crossover complexes [18], ferromagnetic thin films obtained from organic blends [19], and theoretical calculations on 2D porphyrin-based networks for spintronics [20]. We are also happy to host a review paper entitled “Spin-chemistry concepts for spintronics scientists” [21], which discusses the vast terminology differences and addresses the benefits that might arise from a stronger interaction between spin-chemistry and spintronics, thereby opening the horizon for future progress in both fields.

We truly appreciate the open access policy of the Beilstein-Institut, which made this Thematic Series possible. We are greatly indebted to the team at the *Beilstein Journal of Nanotechnology* for their highly professional support and always very fast feedback. We would also like to thank all referees for their effort and constructive criticism.

Georgeta Salvan and Dietrich R. T. Zahn

Chemnitz, October 2017

## References

[1] Aliabadi A, Büchner B, Kataev V, Rüffer T (2017). Beilstein J Nanotechnol.

[2] Abdulmalic M A, Weheabby S, Meva F E, Aliabadi A, Kataev V, Büchner B, Schleife F, Kersting B, Rüffer T (2017). Beilstein J Nanotechnol.

[3] Zaripov R, Vavilova E, Khairuzhdinov I, Salikhov K, Voronkova V, Abdulmalic M A, Meva F E, Weheabby S, Rüffer T, Büchner B (2017). Beilstein J Nanotechnol.

[4] Börner M, Blömer L, Kischel M, Richter P, Salvan G, Zahn D R T, Siles P F, Fuentes M E N, Bufon C C B, Grimm D (2017). Beilstein J Nanotechnol.

[5] Milekhin A G, Cherkasova O, Kuznetsov S A, Milekhin I A, Rodyakina E E, Latyshev A V, Banerjee S, Salvan G, Zahn D R T (2017). Beilstein J Nanotechnol.

[6] Bandari V K, Varadharajan L, Xu L, Jalil A R, Devarajulu M, Siles P F, Zhu F, Schmidt O G (2017). Beilstein J Nanotechnol.

[7] Hahn T, Ludwig T, Timm C, Kortus J (2017). Beilstein J Nanotechnol.

[8] Smykalla L, Mende C, Fronk M, Siles P F, Hietschold M, Salvan G, Zahn D R T, Schmidt O G, Rüffer T, Lang H (2017). Beilstein J Nanotechnol.

[9] Al-Shewiki R K, Mende C, Buschbeck R, Siles P F, Schmidt O G, Rüffer T, Lang H (2017). Beilstein J Nanotechnol.

[10] Waas D, Rückerl F, Knupfer M, Büchner B (2017). Beilstein J Nanotechnol.

[11] Rückerl F, Waas D, Büchner B, Knupfer M, Zahn D R T, Haidu F, Hahn T, Kortus J (2017). Beilstein J Nanotechnol.

[12] Banerjee S, Bülz D, Reuter D, Hiller K, Zahn D R T, Salvan G (2017). Beilstein J Nanotechnol.

[13] Schimmel S, Sun Z, Baumann D, Krylov D, Samoylova N, Popov A, Büchner B, Hess C (2017). Beilstein J Nanotechnol.

[14] Ha N T N, Gopakumar T G, Yen N D C, Mende C, Smykalla L, Schlesinger M, Buschbeck R, Rüffer T, Lang H, Mehring M (2017). Beilstein J Nanotechnol.

[15] Hu G, Xie S, Wang C, Timm C (2017). Beilstein J Nanotechnol.

[16] Chutora T, Redondo J, de la Torre B, Švec M, Jelínek P, Vázquez H (2017). Beilstein J Nanotechnol.

[17] Reichert T, Saragi T P I (2017). Beilstein J Nanotechnol.

[18] Göbel C, Klimm O, Puchtler F, Rosenfeldt S, Förster S, Weber B (2017). Beilstein J Nanotechnol.

[19] Robaschik P, Ma Y, Din S, Heutz S (2017). Beilstein J Nanotechnol.

[20] Tang H, Tarrat N, Langlais V, Wang Y (2017). Beilstein J Nanotechnol.

[21] Ivanov K L, Wagenpfahl A, Deibel C, Matysik J (2017). Beilstein J Nanotechnol.

